# New technologies for identification and surveillance of Chagas disease vectors

**DOI:** 10.1590/0037-8682-0597-2025

**Published:** 2026-03-30

**Authors:** Rodrigo Gurgel-Gonçalves, Genimar Rebouças Julião, Raquel Aparecida Ferreira, Rita de Cássia Moreira de Souza, Mirko Rojas-Cortez, Thallyta Maria Vieira, Vinícius Lima de Miranda, Jonas Lotufo Brant, Marcos Takashi Obara, Rafaella Albuquerque e Silva, Ana Karina Ibarrola-Vannucci, Gerardo Marti, Soledad Ceccarelli

**Affiliations:** 1 Universidade de Brasília, Faculdade de Medicina, Brasília, DF, Brasil.; 2 Fundação Oswaldo Cruz Rondônia, Laboratório de Entomologia, Porto Velho, RO, Brasil.; 3 Fundação Oswaldo Cruz, Instituto René Rachou, Grupo Triatomíneos, Belo Horizonte, MG, Brasil.; 4 Fundación Salud Naturaleza Integral - SANIT, Cochabamba, Bolivia.; 5 Instituto de Salud Global de Barcelona (ISGlobal), España.; 6 Universidade Estadual de Montes Claros, Montes Claros, MG, Brasil.; 7 Universidade de Brasília, Faculdade de Ciências da Saúde, Brasília, DF, Brasil.; 8 Universidade de Brasília, Faculdade de Ciências e Tecnologias em Saúde, Brasília, DF, Brasil.; 9 Ministério da Saúde, Secretaria de Vigilância em Saúde e Ambiente, Departamento de Vigilância das Doenças Transmissíveis, Brasília, DF, Brasil.; 10 Servicio Nacional de Erradicación del Paludismo (SENEPA), Ministerio de Salud Pública y Bienestar Social (MSPBS), Unidad de Proyectos Convenios e Investigación, Asunción, Paraguay.; 11 Instituto Nacional de Salud (INS) - Ministerio de Salud Pública y Bienestar Social (MSPBS), Departamento de Investigaciones, Asunción, Paraguay.; 12 Centro de Estudios Parasitológicos y de Vectores (CEPAVE, CONICET-UNLP-asociado a CIC), La Plata, Buenos Aires, Argentina.

**Keywords:** Chagas disease, Triatomines, Surveillance, Technology, Devices, Apps

## Abstract

Technologies are essential for surveillance of vector-borne diseases. The increasing frequency of triatomine house invasion in the Americas highlights the need to strengthen surveillance strategies. This narrative review examines how emerging technologies can improve identification and reporting of Chagas disease vectors. We analyzed studies published between 2015 and 2025 on digital tools for triatomine surveillance. Technologies were grouped by purpose: (1) identification apps (TriatoKey, TriatoDex, automated identification); (2) community engagement platforms (WhatsBarb, TriatoChat); and (3) institutional surveillance systems (SISVetor-Chagas, GeoVin, Triatomine Information Posts). We summarize their characteristics, applications, knowledge gaps, and potential integration with national surveillance systems, and discuss implications for public health policy. Digital innovation and citizen-based surveillance may support improved prevention and control of vector-borne Chagas disease.

## INTRODUCTION

Chagas disease, caused by the parasite *Trypanosoma cruzi*, is one of the most important vector-borne diseases in the Americas^1^. The parasite is primarily transmitted by triatomine bugs, making vector surveillance central to disease control^2^ ^,^ ^3^. Insecticide spraying has markedly reduced domestic populations of non-native species; however, no comparable reduction has occurred in native species^4^ ^,^ ^5^. In some regions, the abundance and geographic range of native species have increased despite 40 years of vector control programs^6^. The rising frequency of house invasion by triatomines across the Americas^7^ ^-^ ^11^ highlights the need to strengthen surveillance systems for vector reporting and identification.

Digital technologies have become essential for monitoring vectors of neglected tropical diseases^12^. Chagas disease surveillance traditionally relies on household inspections by trained agents, with infestation foci eliminated through insecticide spraying^13^. Detection becomes challenging when vector populations are small, at-risk communities have limited education, or access to referral centers is restricted. Abad-Franch et al.^2^ demonstrated that surveillance is more effective when householders report suspected triatomines than when staff conduct active searches, highlighting the importance of community participation for long-term control. Technology is therefore critical to enabling this approach. 

Researchers have developed digital tools to improve Chagas disease vector identification^14^. Current strategies include online training for health agents, mobile applications for vector identification, citizen science platforms that promote community participation, and transmission risk mapping^15^. Community engagement and vector identification tools can strengthen surveillance^2^ ^,^ ^14^. Despite these advances, a critical gap persists in evaluating and integrating digital technologies that complement community-based surveillance for early detection of low-density triatomine populations across diverse socioenvironmental settings. 

This narrative review examines how emerging technologies can improve triatomine identification and reporting. We searched for studies on triatomine surveillance using the terms “triatomines” and “technology,” “surveillance devices,” “apps,” or “automated identification.” We included original articles, reviews, commentaries, and opinion pieces indexed in PubMed, as well as gray literature, reports, and digital media published in English, Portuguese, or Spanish between 2015 and 2025. Additional references were identified through manual searches. After excluding studies not directly addressing the review focus, 25 articles were analyzed. 

Technologies were grouped by aim: (1) identification apps (TriatoKey, TriatoDex, automated identification); (2) community engagement platforms (WhatsBarb, TriatoChat); and (3) institutional surveillance systems (SISVetor-Chagas, GeoVin, Triatomine Information Posts [TIPs]). We summarize the features and applications of each (Table 1) and discuss their integration with national surveillance systems. These technologies have the potential to strengthen surveillance through community participation and broader societal engagement. 

**TABLE 1:** Target users, validation level, operational applicability, and main limitations of technologies for identification and surveillance of Chagas disease vectors.

Technologies	Target users	Validation level	Operational applicability	Main limitations
TriatoKey	Professionals and general population	Tested by 24 community health agents and 20 ordinary people	Users can send photos to taxonomists or compare suspected insects with available species	Does not include images of all triatomine species native to Brazil
TriatoDex	Professionals	Tested by 27 Brazilian users (reference laboratory workers, surveillance workers, researchers, students)	Includes images of all triatomine species; performed similarly to, but faster than, a printed key	Characters may be insufficient to discriminate among morphologically similar species
Technologies based on machine learning	Professionals and general population	Interface/application under development; not yet validated	Anyone with an internet-connected smartphone can photograph a triatomine, upload the image, and receive identification	Requires incorporation of a greater number of triatomine species
TriatoChat	Professionals and general population	Tested by 10 specialists using a Likert scale; prototype tested with residents of Minas Gerais using a 0-10 satisfaction scale	WhatsApp chatbot provides automated guidance on triatomine identification, Chagas prevention, post-contact procedures, and insecticide use; allows image submission, collects vector occurrence data, and integrates with surveillance systems to generate risk maps	Depends on internet access; accuracy depends on data and image quality; requires continuous validation and integration with official surveillance systems
WhatsBarb	Professionals and general population	Tested in 20 of the 26 states of Brazil and the Federal District	Users can send photos, questions, and comments; responses include insect identification, prevention recommendations, and referral to control and surveillance systems	Volunteer adherence and need for specialist support
SISVetor-Chagas	Professionals	Tested in six municipalities using the System Usability Scale and qualitative assessment	Decentralized architecture allowing municipal, regional, state-level, and federal units to operate independently while maintaining data ownership	High turnover of professionals requiring frequent training; limited financial resources for software sustainability
GeoVin	Mainly general population, also professionals	Tested and used by provincial health agents in 20 of the 23 provinces of Argentina	Users can send photos of suspected triatomines and receive responses coordinated with provincial health authorities	Currently operational only in Argentina
Triatomine Information Posts	General population	Fully operational and validated system	Provides structured community-based insect reception points	Operational applicability currently limited; 20 limitations described in Amaral et al.^35^

APPS TO SUPPORT TRIATOMINE IDENTIFICATION

**FIGURE 1:**
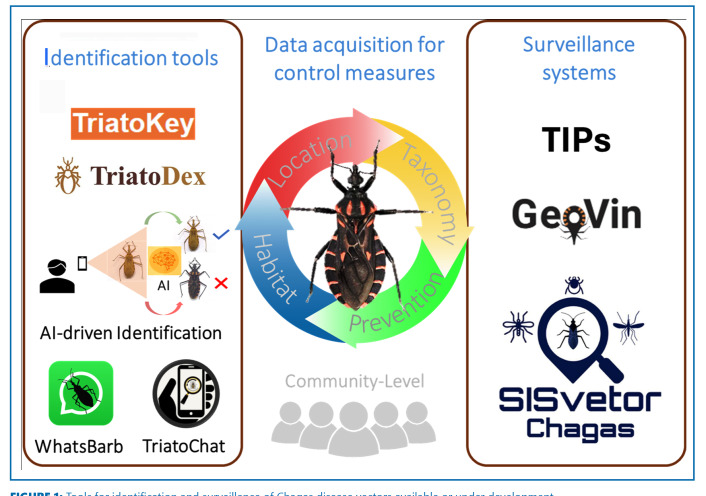
Tools for identification and surveillance of Chagas disease vectors available or under development.

Accurate triatomine identification is essential for Chagas disease surveillance, as species differ in their roles in transmission^16^. In recent years, devices, web platforms, mobile applications, and electronic keys have been developed to facilitate reporting and identification^17^ ^-^ ^20^. Here, we review these tools and summarize their characteristics and applications (Figure 1).

TriatoKey and TriatoDex are electronic identification keys. Automated triatomine identification is under development and may be integrated into surveillance systems alongside electronic keys and community engagement platforms, including the TriatoChat chatbot and WhatsBarb via WhatsApp. Colored arrows represent data flows that strengthen surveillance systems (TIPs, GeoVin, and SISVetor-Chagas) through community participation. These applications can be incorporated into institutional surveillance systems, potentially improving accessibility within surveillance services and enhancing vector monitoring through community engagement.

**AI:** artificial intelligence; **TIPs:** Triatomine Identification Posts.

TriatoKey is a key tool for triatomine identification, particularly in settings where surveillance depends on community participation. Its simple, accessible visual design supports initial identification of suspected insects found inside or around homes. By clearly presenting the general characteristics of triatomines and distinguishing them from other common hemipterans, the app enhances community recognition of potential vectors and promotes timely reporting to health teams^17^. Users can also send photographs of suspected insects directly to the reference service for triatomine identification at the René Rachou Institute (Fiocruz), ensuring rapid and reliable taxonomic confirmation. The tool supports educational activities, local training initiatives, community health agents, and municipal teams, and can be integrated into health campaigns and school programs. Thus, TriatoKey enhances early detection, community engagement, and the efficiency of surveillance systems based on public participation. 

TriatoKey also functions as a practical identification guide for both the general population and surveillance professionals^17^. Using standardized illustrations and concise morphological criteria, it guides users in distinguishing the genera *Panstrongylus*, *Rhodnius*, and *Triatoma* and in identifying their respective species. Its main strength lies in combining operational simplicity with taxonomic rigor. In pilot testing with community health agents and lay users, even those without prior experience were able to distinguish triatomines from other predatory or phytophagous hemipterans and, in many cases, achieve species-level identification among the 42 autochthonous species recorded in Brazil (Table 1). The app is available in English, Spanish, and Portuguese and can be used offline. It also allows electronic submission of georeferenced images when identification is uncertain, enabling taxonomic confirmation at Fiocruz. This feature supports national mapping of vector distribution and improves epidemiological data accuracy. 

To date, more than 2,050 images have been received and analyzed, including 291 triatomines. These were reported from nearly all Brazilian states, except Amapá, the Federal District, and Sergipe. The app has been installed by over 10,000 users, primarily in Brazil but also in the United States, Spain, Peru, South Africa, Argentina, Venezuela, Bolivia, France, and Mexico. TriatoKey is among the most advanced digital tools for triatomine identification, enabling users with varying expertise to achieve consistent results and strengthen vector surveillance systems. Prior to public release, the app was tested and validated with its target audience, including the general population and health agents. The study was approved by the Research Ethics Committee of the Instituto René Rachou (protocol 12930113.0.0000.5091).

TriatoDex is a pictorial, annotated, polytomous key covering 158 triatomine species worldwide^18^. Developed for Android and iOS, it was tested by 27 Brazilian users who identified adult specimens to species level (Table 1). Overall accuracy reached 78.9% across 824 tasks and was higher among trained taxonomists (93.3%). User age, gender, primary job, workplace, and basic training had negligible effects on performance. In a pilot comparison with a printed key, TriatoDex showed similar accuracy but enabled faster identification, saving an average of 2.3 minutes per identification task. It is a practical, flexible, and reliable identification tool. TriatoDex is integrated into the VetorDex app, which includes electronic keys for other vectors and is available on the Apple App Store and Google Play. TriatoDex has been adopted by surveillance services in Brazil and in countries such as Mexico, Colombia, and Peru. Broader use of these technologies may strengthen both entomological surveillance and research on Chagas disease vectors. However, the tool requires prior knowledge for character selection and may not reliably distinguish some morphologically similar species (Table 1). 

Machine learning technologies have been applied to identify triatomine images^21^ ^-^ ^25^, achieving over 90% accuracy at both genus and species levels. Deep learning models show strong performance in identifying Chagas disease vectors^22^, including images captured with mobile phones^19^ ^,^ ^26^. Automated systems can also distinguish triatomines from other Heteroptera species^27^. Expanding image databases is essential to develop open-source mobile applications that provide rapid triatomine identification and strengthen community-based surveillance across the Americas. Users can photograph a suspected insect, upload the image, and receive immediate identification and vector-related information (Table 1). These community-generated data can be stored and automatically integrated into surveillance datasets. 

## DIGITAL PLATFORMS FOR COMMUNITY ENGAGEMENT

TriatoChat was developed in response to misinformation about Chagas disease control and the increasing detection of *T. cruzi*-infected triatomines in urban areas of Montes Claros, Minas Gerais, Brazil^28^. Designed to strengthen health education, support surveillance, and connect science with the community, it aligns with the One Health framework, which integrates populations, health services, and research. The chatbot operates via the WhatsApp messaging platform, eliminating the need for additional applications and enabling rapid, practical access on mobile phones and computers (Table 1). Automated responses provide evidence-based guidance on prevention, insect identification, and appropriate actions following contact with kissing bugs. Beyond information delivery, TriatoChat collects data on triatomine sightings and human-vector contact. These data can be integrated into surveillance systems to generate risk maps and guide control measures. By encouraging public reporting, the platform promotes citizen science and strengthens participatory entomological surveillance. TriatoChat is an accessible and efficient tool that supports triatomine surveillance, Chagas disease prevention, health education, and community engagement. Its implementation enhances awareness, enables continuous vector monitoring, and integrates data and action within a One Health approach, with direct benefits for public health and scientific training. The project was approved by the Research Ethics Committee (protocol 90184025900005148) and tested with the target audience (Table 1). 

WhatsBarb is a citizen surveillance initiative that uses an existing multi-platform application (WhatsApp Business) to integrate community participation into triatomine surveillance and health information services^20^. The strategy involved distributing digital leaflets with images of kissing bugs, scientific names, epidemiological relevance, instructions for insect collection and storage, contact numbers, and delivery points. This approach addressed public demand for identifying triatomine-like insects and clarified related public health concerns^29^. Between 2019 and 2024, 465 insect records were submitted, representing approximately 68 genera, 42 species, and 101 arthropod taxa. Triatomines accounted for 32.3% of reports, while phytophagous bugs and predatory reduviids comprised 54.6% of inquiries. The initiative expanded to 20 of the 26 states of Brazil and the Federal District. WhatsBarb is a rapid, low-cost, user-friendly strategy that enables early identification of risk situations, including in remote areas (Table 1). It supports mapping, prevention, and control of vector-borne diseases and could be adapted to other neglected diseases. Insect records also contribute to biodiversity datasets and taxonomic research. Limitations include variable volunteer engagement, low image quality, incomplete specimens, and limited data fields, highlighting the need for sustained outreach to expand its scope and impact^20^.

## INSTITUTIONAL SURVEILLANCE SYSTEMS FOR TRIATOMINES

The Chagas Disease Control Program Information System, created in the early 1990s, recorded surveillance and control activities in Brazil but is now obsolete and requires replacement. SISVetor-Chagas, developed by the University of Brasília and funded by the Brazilian Ministry of Health, aims to organize entomological surveillance workflows nationwide. It supports management and analysis of entomological data for local decision-making, including defining and managing properties and territories targeted for field interventions, and coordinating related activities and resources. This digital framework strengthens vector control and ensures continuous access to entomological data. 

The system enables ongoing assessment of *T. cruzi* transmission risk and supports macro-level analysis to inform public policy, based on results from individual or integrated localities. It provides a technological platform for transmitting consolidated data on Chagas disease surveillance and vector control actions across the national territory^30^. Currently in a pilot phase in 26 municipalities in Brazil, SISVetor-Chagas will expand to 12 municipalities in the northern region, followed by phased implementation in all municipalities prioritized for acute and chronic Chagas disease.

SISVetor-Chagas was validated in six municipalities in Brazil using the System Usability Scale^31^ and qualitative assessment based on Normalization Process Theory^32^. This process evaluated previously implemented adjustments and informed a continuous improvement plan grounded in the experiences and needs of field users (Table 1). The study was approved by the Ethics Committee of the Faculty of Health Sciences and Technologies, Ceilândia Campus, University of Brasília (protocol 87054324.4.0000.8093). In this preliminary evaluation, 60% of respondents considered the system user-friendly and not requiring prior training, and 70% supported its adoption. Although still in the consolidation phase, SISVetor-Chagas shows strong potential for integration into routine health service practices. Sustained technical and educational support, along with dedicated funding, remains essential to ensure long-term implementation.

The GeoVin project was developed by the Center for Parasitological and Vector Studies in Argentina and collaborators as an open, participatory science initiative^32^. It promotes collection of geographic and habitat data on triatomine sightings to enable timely public health responses in coordination with government agencies. A central tool is a mobile application launched in 2018 to help community members and field technicians identify suspected triatomines and support existing vector control actions (Table 1). Users can submit georeferenced photographs of insects and receive feedback. When a triatomine is confirmed, they are advised on how to contact local vector control services. To strengthen collaboration between health authorities and communities, management of the response control panel was decentralized three years ago. Each province in Argentina can now designate a technical or scientific team to access and respond to reports.

The GeoVin app has recorded over 3,500 downloads, about 2,200 registered users, and more than 3,000 reports by 2025. Although higher numbers of triatomine reports are expected in endemic areas, many submissions originated from major cities and surrounding regions. This pattern likely reflects differences in outreach intensity and communication strategies, as well as social media activities led by the working group in Buenos Aires province. Approximately 85% of reports involved non-triatomine insects. By identifying these specimens and clarifying that they were not kissing bugs, the team provided reassurance to community members^33^.

In Brazil, community-based Chagas disease surveillance relies primarily on TIPs as a core strategy^34^. These posts are officially designated sites for receiving insects suspected to be triatomines^32^. Effective operation in partnership with communities is critical for successful surveillance. A study conducted in high-risk areas of Minas Gerais assessed gaps in TIP functioning and efficiency^35^. Approved by the Research Ethics Committee of the René Rachou Institute under approval number 37132220.8.0000.5091, the study identified 20 key barriers to TIP maintenance. These findings informed development of a protocol to sustain a municipal TIP network by identifying operational gaps, guiding corrective actions, and strengthening long-term sustainability. Despite their importance, Brazil lacks monitoring tools for TIPs. 

To ensure nationwide public health coverage, similar assessments must extend to low- and medium-risk areas. A second study is underway in additional risk-stratified regions of Minas Gerais to broaden understanding of sustainability challenges. This project was approved by the same ethics committee under approval number 83611524.9.3001.5651. The protocol was subsequently expanded and field-validated. It represents an innovative tool to map circulating vector profiles in low-density settings and areas with presumed absence of transmission, thereby supporting public policy planning (Table 1). Integration of this validated tool into the SISVetor module is planned; development and operational design remain in progress. 

The Brazilian Chagas disease website of the Oswaldo Cruz Foundation centralizes and disseminates information using digital and educational tools. It provides three options for reporting suspected triatomines: submitting a photograph through an online form, delivering the specimen in person via PITsMaps, or identifying it through a link to TriatoKey. PITsMaps enables users to access addresses, telephone numbers, and contact details of TIPs across Brazil^36^. However, this approach may be challenging in settings with high turnover among entomological surveillance personnel and within the TIP network. This platform exemplifies integration of the technological tools described in this review.

## CONCLUSIONS AND OUTLOOKS

Detecting residual infestation foci or reinfestation in homes remains a major challenge for entomological surveillance of Chagas disease in the Americas. Current recommendations emphasize community-led surveillance, continuously implemented and territorially integrated, and supported by priority health policies to ensure stability despite institutional or political changes. This model has been widely applied in citizen science initiatives targeting mosquitoes^37^ ^,^ ^38^ and ticks^39^. Citizen science also provides reliable data on triatomine distribution^40^ ^-^ ^43^. Websites, email, mobile applications, social media, and other digital tools facilitate large-scale, long-term surveillance through community participation^44^ ^,^ ^45^. For instance, Hill et al.^46^ demonstrated that iNaturalist, a citizen science tool, can help address gaps in triatomine distribution data. 

Digital technologies for triatomine identification and biogeographic mapping are essential to strengthen national entomological surveillance systems. However, development remains uneven across the region. Brazil, for example, has implemented multiple technological applications, reflecting a diverse and advanced landscape. The current priority is integrating these tools or ensuring interoperability to enable seamless data exchange within and between countries. Such integration would facilitate technology transfer to countries that have not yet developed these systems or remain in early implementation stages. Sustainability depends on accessibility, free use, simplicity, and user engagement for community members, field staff, and students. Pilot projects across countries demonstrate the feasibility of these tools as effective support mechanisms for regional surveillance systems. These experiences are being consolidated by the Global Triatomine Network Initiative, which brings together public health institutions, academics, and non-governmental organizations across the Americas to advance these objectives.

The Global Network for the Control of Triatomine Bugs Involved in Chagas Disease (RedTri) was established to strengthen existing partnerships and engage new institutions to improve integration of triatomine-related data into regional control programs. A key outcome is the development of technological tools for visualizing updated information and for surveying and identifying triatomines, recognized as essential to interrupting vector-borne transmission of *T. cruzi*, the causative agent of Chagas disease. RedTri also promotes technology transfer and collaboration, including mobile applications for reporting and identification and artificial intelligence systems for automated triatomine recognition. Network members have produced peer-reviewed papers and books on entomology, artificial intelligence, and citizen science, ensuring scientific rigor. The initiative has gained regional relevance, planning GeoVin pilot studies in Brazil, Ecuador, and Mexico for 2026 and establishing agreements with national institutions such as CeNDIE (ANLIS Malbrán, Ministry of Health of Argentina) and international partners including Fiocruz Minas in Brazil. 

Digital technologies are central to modern disease surveillance. This review did not compare current tools for Chagas disease vector surveillance by performance, coverage, scalability, or cost. As shown in the text and Table 1, these technologies differ in objectives and stages of implementation and maturity, precluding direct comparison. Instead, we summarized target users, validation outcomes, operational applicability, and key limitations. Emerging approaches used in digital entomological surveillance for other vectors may inform future applications. These include: (1) autonomous traps with sensors, cameras, and connectivity for proactive real-time vector and environmental monitoring; (2) global participatory platforms enabling public submission of georeferenced images to expand spatial and temporal coverage and support big data modeling and early warning systems; (3) acoustic classification using species-specific sound signatures, such as wing-beat frequencies, for real-time identification; (4) integrated digital dashboards combining entomological, epidemiological, and operational data to guide rapid response; and (5) satellite imagery, remote sensing, drones, and spatial modeling to map vector distribution and optimize field resource allocation^41^ ^,^ ^47^ ^-^ ^51^. Although not yet widely applied to Chagas disease, these innovations show promise and warrant adaptation and evaluation to strengthen surveillance and vector control in endemic settings. 

The use of digital technologies for Chagas disease vector surveillance raises key ethical and governance issues^52^. These include protection of georeferenced data, management of community-submitted information, recognition of volunteer contributions, and clear communication of collaboration expectations. As systems increasingly rely on cloud-based platforms and cross-border data sharing, robust governance frameworks are essential to address data sovereignty and ensure compliance with national and international public health regulations. For example, the GeoVin app includes an explicit data policy that users must read and accept.

In conclusion, this review summarized the target users, validation, operational applicability, and main limitations of triatomine identification tools (TriatoKey, TriatoDex, automated identification), community engagement platforms (WhatsBarb, TriatoChat), and institutional surveillance systems (SISVetor-Chagas, GeoVin, TIPs). Technology and citizen surveillance have strong potential to inform and improve public policies for prevention and control of vector-borne Chagas disease.
